# Factors associated with mortality in patients with super-refractory status epilepticus

**DOI:** 10.1038/s41598-022-13726-9

**Published:** 2022-06-11

**Authors:** Yi-Ting Fang, Tsung-Lin Lee, Yi-Hsien Tu, Sheng-Hsiang Lin, Miao-Er Chien, Chin-Wei Huang, Kuei-Sen Hsu, Yi-Jen Wu

**Affiliations:** 1 Department of Neurology, National Cheng Kung University Hospital, College of Medicine, National Cheng Kung University, Tainan, 70403 Taiwan; 2 Department of Neurology, E-Da Hospital, Kaohsiung, 82445 Taiwan; 3 Institute of Clinical Medicine, College of Medicine, National Cheng Kung University, No. 35, Xiaodong Rd., Tainan City, 70457 Taiwan; 4 Biostatistics Consulting Center, National Cheng Kung University Hospital, College of Medicine, National Cheng Kung University, Tainan, 70457 Taiwan; 5 Department of Pharmacology, College of Medicine, National Cheng Kung University, Tainan, 70101 Taiwan; 6 Institute of Basic Medical Sciences, College of Medicine, National Cheng Kung University, Tainan, 70101 Taiwan

**Keywords:** Epilepsy, Neurology, Neurological disorders

## Abstract

Super-refractory status epilepticus (SRSE) is a critical condition in which seizures persist despite anesthetic use for 24 h or longer. High mortality has been reported in patients with SRSE, but the cause of death remains unclear. We investigated the factors associated with mortality, including clinical characteristics, SE etiologies and severities, treatments, and responses in patients with SRSE in a 13-year tertiary hospital-based retrospective cohort study comparing these parameters between deceased and surviving patients. SRSE accounted for 14.2% of patients with status epilepticus, and 28.6% of SRSE patients died. Deceased patients were mostly young or middle-aged without known systemic diseases or epilepsy. All deceased patients experienced generalized convulsive status epilepticus and failure of anesthetic tapering-off, significantly higher than survivors. An increased number of second-line anesthetics besides midazolam was observed in the deceased (median, 3, interquartile range 2–3) compared to surviving (1, 1–1; *p* = 0.0006) patients with prolonged use durations (*p* = 0.047). For mortality, the cut-off number of second-line anesthetics was 1.5 (AUC = 0.906, *p* = 0.004). Deceased patients had significantly higher renal and cardiac complications and metabolic acidosis than survivors. In SRSE management, multi-anesthetic use should be carefully controlled to avoid systemic complications and mortality.

## Introduction

Status epilepticus (SE) is a critical state of prolonged, repeated seizures when the brain loses seizure termination mechanisms^1^. SE is a neurological emergency as it can progress into sustained epileptic seizures when the seizure lasts for over 5 min and can have long-lasting consequences when it continues for over 30 min in the convulsive SE^2^. Current guidelines suggest several pharmacological strategies for treating SE. Benzodiazepines are initially administered at the early pre-hospital stages of seizures. Intravenous (IV) anti-seizure medications (ASMs) are given after arrival at the hospital in the early stages of the established SE. If seizures cannot be controlled by IV ASM administration, general anesthesia is subsequently adopted for refractory SE, e.g., by IV infusion of midazolam, propofol, or ketamine^3,4^. Anesthetics are continuously infused until the seizure subsides and is maintained for 12–24 h following the last seizure^5^. If SE continues or recurs despite using anesthetics for 24 h or longer, it is defined as super-refractory SE (SRSE)^6^. The incidence rate of SRSE was reported as 3 in 100,000 per year and accounted for 13.1% of SE events in a German population-based study^7^, whereas 0.7 in 100,000 per year and 5–10% of SE were determined in data from a Finnish Intensive Care Consortium^8^. It has been estimated that around 15% of all hospital-admitted cases with SE progress into SRSE with mortality rates of 30–50%^6^. SRSE mortality rates vary around 40% among different cohorts; 42.1% was reported in a French teaching hospital-based 6-year cohort study^9^, 37.9% in a Swiss hospital-based 9-year cohort study^10^, and 39.9% in a German health insurance database 6-year cohort study. These mortality rates are significantly higher than those in non-refractory SE (9.6%)^7^. However, the risk factors associated with mortality in SRSE remain unclear. *Objectives* In the present retrospective study, the baseline characteristics of patients with SRSE, their SE etiologies and severities, and SRSE treatments and responses were analyzed. These clinical factors and treatment outcomes were compared between deceased and surviving patients within a homogenous SRSE cohort from a tertiary teaching hospital to identify factors associated with mortality in patients with SRSE.

## Materials and methods

### Study design and cohort

#### Setting

This retrospective observational cohort study enrolled patients with SRSE admitted to the neurology department between January 2007 and December 2019 at the National Cheng Kung University Hospital. This study obtained ethical approval for research with human participants from the Institutional Review Board, National Cheng Kung University Hospital (IRB No: B-ER-107-106). Waiver of informed consent was granted by the Institutional Review Board of National Cheng Kung University Hospital. All procedures were performed in accordance with the relevant guidelines and regulations.

#### Participants

First, patients with SE were screened from the hospital inpatient database with admission or discharge diagnosis of status epilepticus. SE was defined as repeated seizures lasting > 30 min^2^. In contrast to the common definition of SE as a seizure lasting > 30 min, International League Against Epilepsy (ILAE) 2015 redefined SE as t1 when a seizure is likely to become prolonged and sustained, and t2 when a seizure will potentially cause long term consequences^2^. Tonic–clonic SE was defined with 5 min as t1 and 30 min as t2, while focal SE with impaired consciousness was defined as 10 min as t1 and > 60 min as t2. Although the recent ILAE definition indicates the SE can be identified earlier than 30 min, we adopted the common definition of SE for both convulsive and non-convulsive SE as seizures lasting > 30 min or the consciousness did not recover within the repeated seizures for more than 30 min, respectively, to cover the long recruitment period despite the evolution of definition across time. The medical records were reviewed to identify patients with SRSE, defined as clinical or electrographic seizures despite the continuous infusion of anesthetics such as midazolam and other secondary anesthetics for 24 h or longer. Patients with SE who did not receive anesthetics or those with seizures that anesthetics within 24 h could control were excluded from the following SRSE analysis. *Variables* The baseline characteristics of the enrolled patients with SRSE, including age, sex, systemic diseases, and known epilepsy, were extracted from medical records. The number of days of hospital and intensive care unit (ICU) stay was also documented. The etiologies of SE were based on the ILAE 2015 Task Force report^2^. The presence of cerebral trauma, tumor, infection, vascular disorders, immunological disorders, neurodegenerative diseases, or seizure-provoking factors, including fever, metabolic or endocrine disorders, drug or alcohol abuse, toxins, menstrual cycle, and sleep–wake problems, were defined as etiology and documented. Data from initial event-related laboratory examinations, such as brain imaging, cerebrospinal fluid (CSF) and serum analyses, including blood cell count, prolactin level, and pH, were collected. The SE classification was based on the ILAE 2015 report^2^ to sort our cohort into generalized convulsive SE, focal motor SE and nonconvulsive SE. Consciousness was scored using the Glasgow Coma Scale (GCS) when the patient arrived at the emergency department. The overall SE severity was assessed using the Status Epilepticus Severity Score (STESS), comprising data on consciousness, age, most severe seizure type, and history of previous seizures with a total score of 6, among which a favorable STESS score of 0–2 indicates a low mortality risk for patients with SE according to the study by Rossetti et al.^11^.

### Treatment and response

Since adequate early treatment affects SE outcomes^12^, we documented door-to-needle times and treatment durations of IV infusions with diazepam, ASMs, midazolam, and second-line anesthetics, defined as the treatment for SRSE in the study. Under these treatments, response was determined following 24-h anesthetic use by measuring seizure control. Door-to-treatment time was defined as the latency between treatment administration and arrival at the hospital. Intravenous ASM numbers and door-to-first IV ASM times were reviewed, and the differences between deceased and surviving patients with SRSE were compared. When seizures failed to be controlled by IV ASMs, anesthetics were subsequently applied. In accordance with treatment guidelines^13^, midazolam IV was the first anesthetic to treat refractory SE in our clinical practice. Second-line anesthetics were defined as anesthetics other than midazolam, which were prescribed when midazolam failed to control seizures. The duration of second-line anesthetic use was defined as the total time, per patient, of all the anesthetic drugs, excluding midazolam. Recently, the debate regarding anesthetics in the treatment of refractory SE has focused on the risk–benefit uncertainty due to prolonged hospital stay in patients with anesthetic use^14^. Therefore, we determined the numbers, door-to-treatment times, and duration of the anesthetics and compared them between the deceased and surviving patients with SRSE to investigate whether anesthetic use affected SRSE complications and mortality rates. Regarding the simultaneously combined use of multiple anesthetics, the durations of each anesthetic were displayed aligned with the admission period of each patient, and the overlapping periods of all anesthetics, including midazolam and second-line anesthetics, were summed and compared between groups. The patients with SRSE were all admitted to the ICU with continuous electroencephalography (EEG) and close clinical/behavior observation to monitor seizures. Treatment responses following 24-h anesthetic use as well as the ASMs and all in-hospital treatments, were evaluated based on the continuous-EEG and clinical responses. They were defined as the (1) remaining clinical seizures, (2) electrographic seizures without clinical manifestation, (3) EEG burst suppression without seizures, and longer after 24-h during the tapering period (4) whether the failure of anesthetic tapering-off following a period of anesthetic use. In our general clinical practice and in agreement with the National Institute for Health and Care Excellence (NICE) treatment guideline^3^, the anesthetic was gradually tapered off after a seizure-free interval for 12–24 h. A failure in anesthetic tapering-off was defined as the occurrence of clinical breakthrough or electrographic seizures during or after the anesthetic had been tapered off. Cardiac, renal, and hepatic dysfunctions that emerged during admission were reviewed and compared between the two study groups to examine systemic complications of patients with SRSE.

### Statistical analysis

Results are presented as the median and interquartile range (IQR) or the number of patients (n) and percentage. Significance levels of differences between the two study groups were calculated using Fisher’s exact test for categorical data and the Mann–Whitney U test for independent numerical variables. Discrimination was defined as the ability of the number of second-line anesthetics to differentiate between deceased and surviving patients based on the receiver operating characteristic (ROC) curve. The statistical significance level was set at *p* < 0.05. All statistical analyses were performed using the Prism 6 software package (GraphPad Software Inc.).

### Ethics approval

This study obtained human study ethical approval (IRB No: B-ER-107-106) from the Institutional Review Board, National Cheng Kung University Hospital, 138 Sheng-Li Rd, Tainan 704, Taiwan.

### Consent to participate

Waiver of informed consent was granted by the IRB.

## Results

### Characteristics, laboratory test results, SE etiologies and severities in patients with SRSE

**Figure 1:**
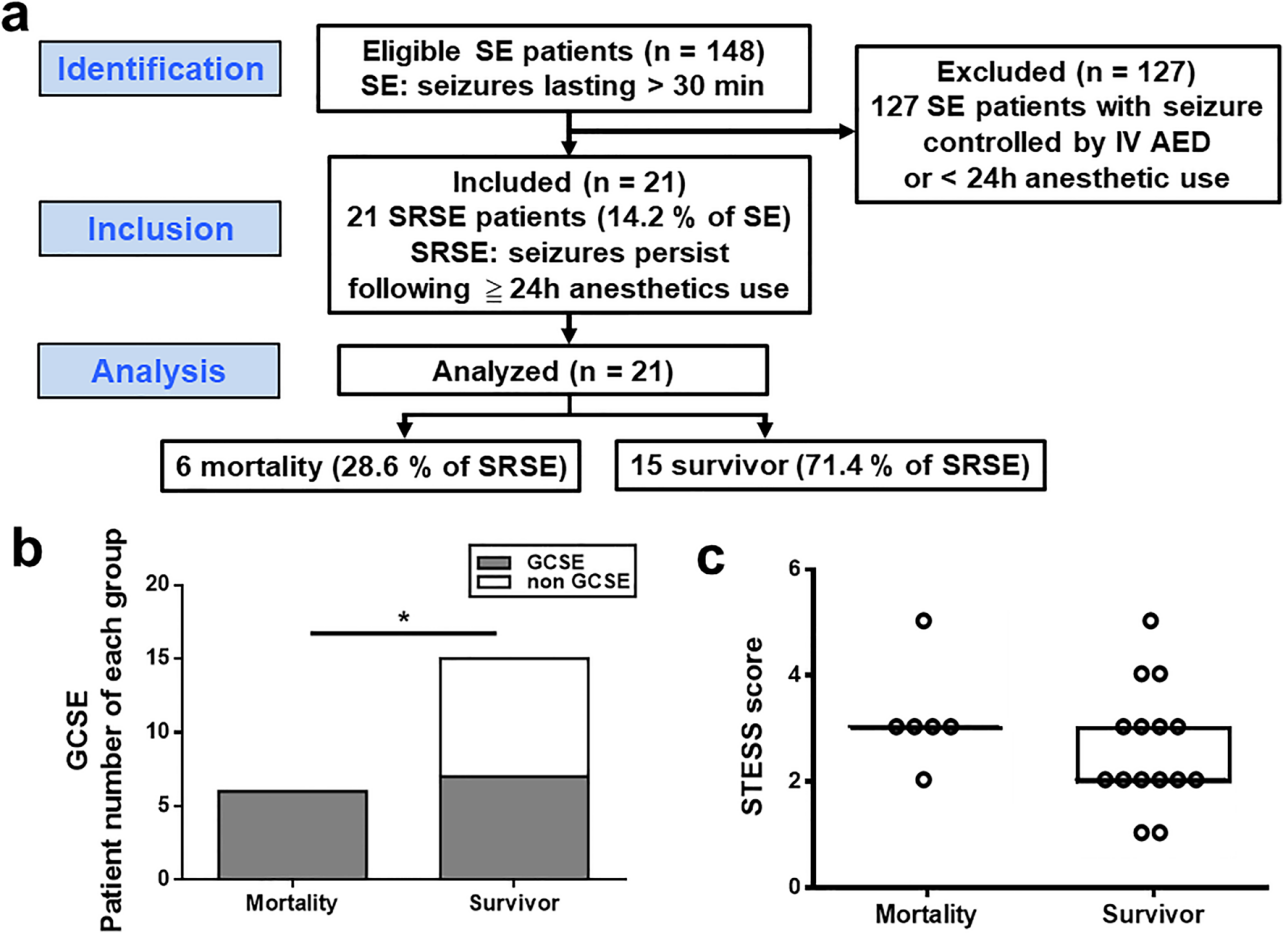
Patient distribution, initial SE type, and SE severity in patients with SRSE. (**a**) Study flowchart in line with STROBE statement and the patient distributions in the mortality and survival groups. (**b**) Numbers of patients with generalized convulsive SE in the mortality and survival groups at hospital admission. (**c**) Initial SE severity was assessed using the STESS in the mortality and survival groups. *p < 0.05. Dark lines indicate median values, and boxes indicate interquartile ranges in (**c**). *SE* status epilepticus, *SRSE* super-refractory status epilepticus, *GCSE* generalized convulsive status epilepticus, *STESS* status epilepticus severity score. *STROBE* strengthening the reporting of observational studies in epidemiology.

A total of 148 patients with SE were identified in this 13-year tertiary hospital-based cohort study, and 21 patients with SRSE were identified in this group (14.2%). Of those, 6 patients with SRSE died during the hospital stay, and 15 survived. The mortality and survival rates among patients with SRSE were 28.6% and 71.4%, respectively, at discharge (Fig. 1a). There was no significant difference in age (*p* = 0.126) and sex (*p* > 0.99) between mortality (M) and survival (S) groups. However, a trend toward younger age was observed in deceased (median 28, IQR 19–43 years) compared to surviving (55, 32–68 years; Table 1) patients. Pre-hospital systemic diseases were present in 66.7% of the survivors and were significantly higher than the corresponding percentage in deceased patients (0%, *p* = 0.012, Fisher’s exact test). Likewise, 40% of the survivors had an epilepsy diagnosis before admission, whereas none of the deceased patients had epilepsy prior to hospitalization. The duration of hospital (median, M 21.5 vs. S 33.0 d, *p* = 0.117, Mann–Whitney U test) and ICU stay (median, M 21.5 vs. S 24.0 d, *p* = 0.492, Mann–Whitney U test) were not significantly different between groups. All patients of the M group had died while being treated in the ICU, whereas those of the S group were successfully tapered off the anesthetics and transferred to the general ward after ICU care. Among the six patients in the M group, four patients were diagnosed with central nervous system (CNS) infection (bacterial or viral meningoencephalitis) and one with CNS immunological disorder (Hashimoto’s encephalopathy; Table 1). Fever was one of the common provoking or associated factors, which can also be a symptom reflecting the underlying SE etiology and preceding seizures in 4 (3 CNS infections and 1 fever of unknown etiology) out of 6 patients in the M group. Among the 15 survivors, 1 patient had head trauma, 4 had CNS infection (3 bacterial or viral meningoencephalitis and 1 prion disease), 4 had cerebrovascular disease (1 intracranial hemorrhage, 1 subdural hemorrhage, and 2 ischemic strokes), and 1 degenerative disease (parkinsonism and vascular dementia). Eleven patients with provoking factors included 1 alcohol withdrawal, 1 hypoxic encephalopathy, and 1 sepsis, which can represent specific etiologies of SE per se, and 8 with fever. Three deceased patients and 7 survivors had existing etiologies and superimposed acute provoking factors, with fever as the most common, attributing to SE in the cohort. Serum leukocytosis and elevated prolactin levels were commonly seen in both groups. In contrast, acidemia and abnormal CSF findings (elevated white blood cell count and protein levels) were more frequently observed in the M groups patients when they arrived at the emergency department (Table 1). Generalized convulsive SE was observed in all patients of the M group (100.0%), which was a significantly higher percentage than that in the S group (46.7%, *p* = 0.046, Fisher’s exact test; Fig. 1b). Among survivors, 40% had focal motor SE, and 13.3% had nonconvulsive SE (Table 1). Consciousness scores based on the GCS (median, IQR, M 7, 6–8 vs. S 4, 3–8, *p* = 0.313, Mann–Whitney U test) and STESS assessing SE severity (median, IQR, M 3, 3–3 vs. S 2, 2–3, *p* = 0.261, Mann–Whitney U test) were not significantly different between the two groups when patients arrived at the emergency room (Fig. 1c).

**Table 1:** Characteristics, laboratory findings, SE etiologies and severities in patients with SRSE.

	Mortality n = 6	Survival n = 15	*p* value
**Patient characteristics**			
Age, median (IQR)	28 (19–43)	55 (32–68)	0.126
Sex, n (%)	M = 3 (50.0%) F = 3 (50.0%)	M = 8 (53.3%) F = 7 (46.7%)	> 0.99
Systemic disease, n (%)	0 (0%)	10 (66.7%)	0.012*
Epilepsy, n (%)	0 (0%)	6 (40.0%)	0.123
Hospital stay (d)	21.5 (6–30)	33.0 (26–69)	0.117
ICU stay (d)	21.5 (6–30)	24.0 (16–42)	0.492
**Etiology**			
Cerebral trauma, n (%)	0 (0%)	1 (6.7%)	> 0.99
Cerebral tumor, n (%)	0 (0%)	0 (0%)	> 0.99
Cerebral infection, n (%)	4 (80.0%)	4 (26.7%)	0.146
Cerebrovascular disorders, n (%)	0 (0%)	4 (26.7%)	0.281
Cerebral immunologic disorders, n (%)	1 (16.7%)	0 (0%)	0.286
Degenerative and other neurologic conditions, n (%)	0 (0%)	1 (6.7%)	> 0.99
Provoking factors as etiology, n (%)	4 (60.0%)	11 (85.7%)	> 0.99
**Initial laboratory findings**			
Brain structure lesion, n (%)	0 (0%)	6 (40.0%)	0.123
CSF abnormalities, n (%)	5 (83.3%)	3^†^ (n = 9, 33.3%)	n/a
Acidemia, n (%)	3^†^ (n = 4, 75%)	5^†^ (n = 12, 41.7%)	n/a
Leukocytosis, n (%)	6 (100.0%)	12 (80.0%)	0.530
Elevated prolactin level, n (%)	4^†^ (n = 4, 100.0%)	9^†^ (n = 9, 100.0%)	n/a
**SE semiology and clinical severity scores**			
Generalized convulsive SE, n (%)	6 (100.0%)	7 (46.7%)	0.046*
Focal motor SE, n (%)	0 (0%)	6 (40.0%)	0.123
Nonconvulsive SE, n (%)	0 (0%)	2 (13.3%)	> 0.99
GCS, median (IQR)	7 (6–8)	4 (3–8)	0.313
STESS, median (IQR)	3 (3–3)	2 (2–3)	0.261

*n* number, *F* female, *M* male, *IQR* interquartile range, *ICU* intensive care unit, *CSF* cerebrospinal fluid, *SE* status epilepticus, *SRSE* super-refractory status epilepticus, *GCS* Glasgow Coma Scale, *STESS* Status Epilepticus Severity Score, *n/a* not applicable due to missing data. ^†^missing data. *p < 0.05.

### Treatments and responses in patients with SRSE

**Figure 2:**
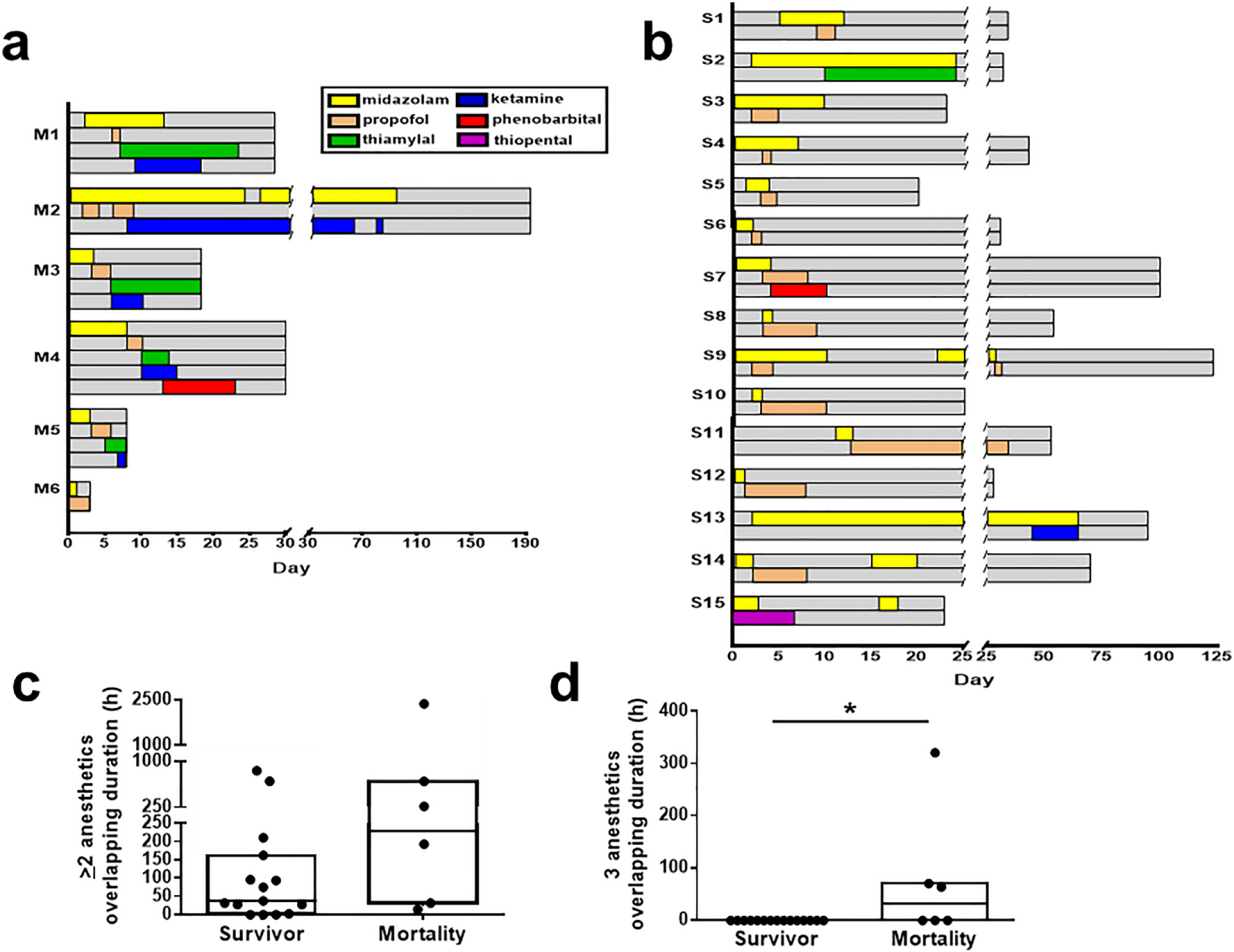
Time courses of anesthetics used in patients with SRSE. (**a**) Time courses of anesthetics used in patients of the mortality group following their hospital admission. (**b**) Time courses of anesthetics used in surviving patients with SRSE. (**c**) Overlapping durations of the combined use of two or more anesthetics comparing survival and mortality groups. (**d**) Overlapping durations of the combined use of three anesthetics comparing survival and mortality groups. Gray bars indicate the total duration of hospital stay for each patient. Anesthetics are labeled in different colors and presented chronologically for the hospital stay. *SRSE* super-refractory status epilepticus.

**Figure 3:**
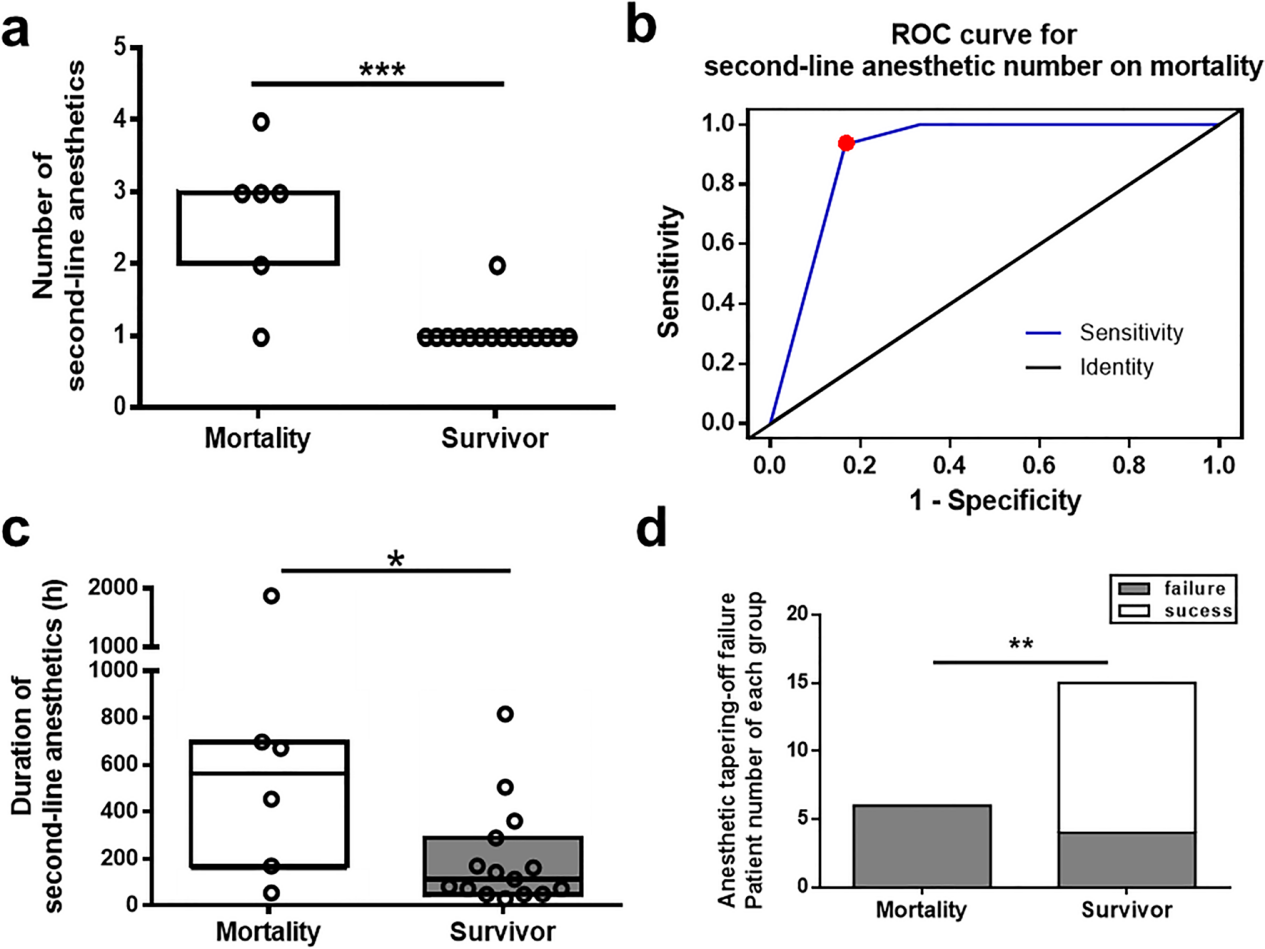
Second-line anesthetic use in patients with SRSE. (**a**) Numbers of second-line anesthetics used in the mortality and survival groups. (**b**) ROC curve for the number of second-line anesthetics to discriminate mortality. (**c**) Duration of second-line anesthetic use in mortality and survival groups. (**d**) Patient numbers for success or failure of tapering off anesthetics in the mortality and survival groups. *p < 0.01, **p < 0.01, ***p < 0.01. Dark lines indicate median values, and boxes indicate interquartile ranges (**a**) and (**c**)*.* The red dot in (**b**) indicates the cutoff point of 1.5. *SRSE* super-refractory status epilepticus, *ROC* receiver operating characteristic.

All patients enrolled in this study received diazepam, IV ASM, midazolam, and second-line anesthetics. First-line IV diazepam was administered within 30 min after arriving at the emergency room in more than 50% of patients in both M and S groups. Following diazepam, both patient groups received 2–3 IV ASMs such as valproic acid, phenytoin, and levetiracetam without significant differences in IV ASM numbers (median, M 3 vs. S 2, *p* = 0.580, Mann–Whitney U test) and door-to-first IV ASM time (median, M 2 vs. S 1.5 h, *p* = 0.957, Mann–Whitney U test; Table 2). The first anesthetic, midazolam, was prescribed following IV ASM use because the seizures persisted (door-to-midazolam time, median, M 3 vs. S 13 h, *p* = 0.258, Mann–Whitney U test) and continued for days (median, M 98 vs. S 65 h, *p* > 0.99, Mann–Whitney U test) without significant intergroup differences. Second-line anesthetics, propofol, ketamine, phenobarbital, thiopental, and thiamylal sodium, were administered when midazolam failed to control seizures (Fig. 2a,b). Second-line anesthetics were added-on to midazolam [patient numbers (%), M 2 (33.3% in M) vs. S 6 (40% in S)] or replaced midazolam [M 4 (66.7% in M) vs. S 9 (60% in S)] in both groups. Add-on of second-line anesthetics was defined when it was initiated after midazolam and with overlapping duration under midazolam use (patient ID, M1, M2, S1, S2, S3, S4, S9, S13). The replacement was defined as when midazolam was discontinued after applying the second-line anesthetics (patient ID, M3, M4, M5, M6, S5, S6, S7, S8, S10, S11, S12, S14, S15), as shown in Fig. 2a,b. Propofol was the most common first choice as the second-line anesthetics in both groups (patient numbers, M 6 vs. S 12). The median initiation time of the second-line anesthetic was 51 h in the M group and 62 h in the S group (*p* = 0.390, Mann–Whitney U test). The maximal infusion dosage of the second-line anesthetic varied for propofol (M 38.3–200 vs. S 33–166 mcg/kg/min) and ketamine (M 0.07–5.0 vs. S 0.27 mg/kg/h) in both groups with the referenced range of propofol 20–200 mcg/kg/min and ketamine 1–10 mg/kg/h for refractory SE^15^. There were no significant differences between M and S groups when comparing the maximal infusion dosage of midazolam (median, M 1.95 vs. S 2, *p* = 0.356, Mann–Whitney U test) and propofol (median, M 152.5 vs. S 93.33, *p* = 0.184, Mann–Whitney U test; Supplementary Figure). Multiple anesthetics used during the hospital stay were more frequently observed in the M group than in the S group. The sum of simultaneously combined use durations of two or more anesthetics (midazolam with at least one second-line anesthetic) was comparatively increased in the M group (Fig. 2c). When analyzing the combined use of three anesthetics (midazolam with two second-line anesthetics), the M group had a longer duration of simultaneous anesthetic use than the S group (*p* = 0.015; Fig. 2d). The number of second-line anesthetics used was also significantly higher in the M group than in the S group (median, IQR: 3, 2–3 vs. 1, 1–1, respectively; *p* = 0.0006, Mann–Whitney U test; Fig. 3a). The ROC curve for the number of second-line anesthetics was analyzed regarding its ability to discriminate M from S group patients. The optimal cut-off point for the number of second-line anesthetics was 1.5 (AUC = 0.906, *p* = 0.004; sensitivity = 0.933, 95% confidence interval [CI] = 0.681 to 0.998, specificity = 0.833, 95% CI = 0.359 to 0.996, likelihood ratio 5.6; Fig. 3b). The summed durations of all second-line anesthetics were increased in M group patients (median, IQR 561, 168–669 h) compared to survivors (112, 48–288 h; *p* = 0.047, Mann–Whitney U test; Fig. 3c). Following the first 24-h treatment, four out of six (66.7%) M group patients had clinical seizures, and two patients (33.3%) achieved EEG burst suppression under anesthetics. Three S group patients (20%) had clinical seizures, five (33.3%) presented nonconvulsive electrographic seizures, five (33.3%) achieved EEG burst suppression, and two (13.3%) had slow waves on EEG without clinical seizures (Table 2). The failure rate when tapering off the anesthetics was significantly higher in the M group (100.0%) than in the S group (26.7%, *p* = 0.004, Fisher’s exact test; Fig. 3d). Besides ASM and anesthetics, immunotherapy such as intravenous methylprednisolone was applied to treat patients with SRSE in the cohort (patient numbers (ID, %), M 3 (M1, M2, M3, 50%) vs. S 2 (S2, S3, 13.3%), and one patient (M1) received double filtration plasmapheresis and plasma exchange.

**Table 2:** Treatments, responses, and complications in patients with SRSE.

	Mortality n = 6	Survival n = 15	*p* value
**Pharmacological treatments**			
Diazepam < 30 min, n (%)	3 (50.0%)	10 (66.7%)	0.631
IV ASM number	3 (2–3)	2 (2–3)	0.580
Door-to-1st IV ASM time, h, median (IQR)	2 (1.0–24.0)	1.5 (1.0–11.8)	0.957
Door-to-midazolam time, h, median (IQR)	3 (1.4–9.8)	13 (1.5–27.0)	0.258
Duration of midazolam use, h, median (IQR)	98 (24.0–240.0)	65 (33.5–216.0)	> 0.99
Door-to-second-line anesthetic time, h, median (IQR)	51 (17.6–96.0)	62 (47.5–120.0)	0.390
Number of second-line anesthetics, median (IQR)	3 (2–3)	1 (1–1)	0.0006***
Duration of second-line anesthetic use, h, median (IQR)	561 (168–669)	112 (48–288)	0.047*
**Treatment responses**			
Clinical seizure after 24-h treatment	4 (66.7%)	3 (20.0%)	0.120
Electrographic seizure after 24-h treatment	0 (0%)	5 (33.3%)	0.262
EEG burst suppression after 24-h treatment	2 (33.3%)	5 (33.3%)	> 0.99
Failure of anesthetic tapering	6 (100.0%)	4 (26.7%)	0.004**
**Complications**			
Renal dysfunction, n (%)	5 (83.3%)	2 (13.3%)	0.006**
Cardiac dysfunction, n (%)	6 (100.0%)	4 (26.7%)	0.004**
Hepatic dysfunction, n (%)	4 (66.7%)	6 (40.0%)	0.361
Metabolic acidosis, n (%)	6 (100.0%)	4 (26.7%)	0.004**

*n* number, *IQR* interquartile range, *IV ASM* intravenous anti-seizure medication, *SRSE* super-refractory status epilepticus. *p < 0.05. **p < 0.01. ***p < 0.001.

### Complications

**Figure 4:**
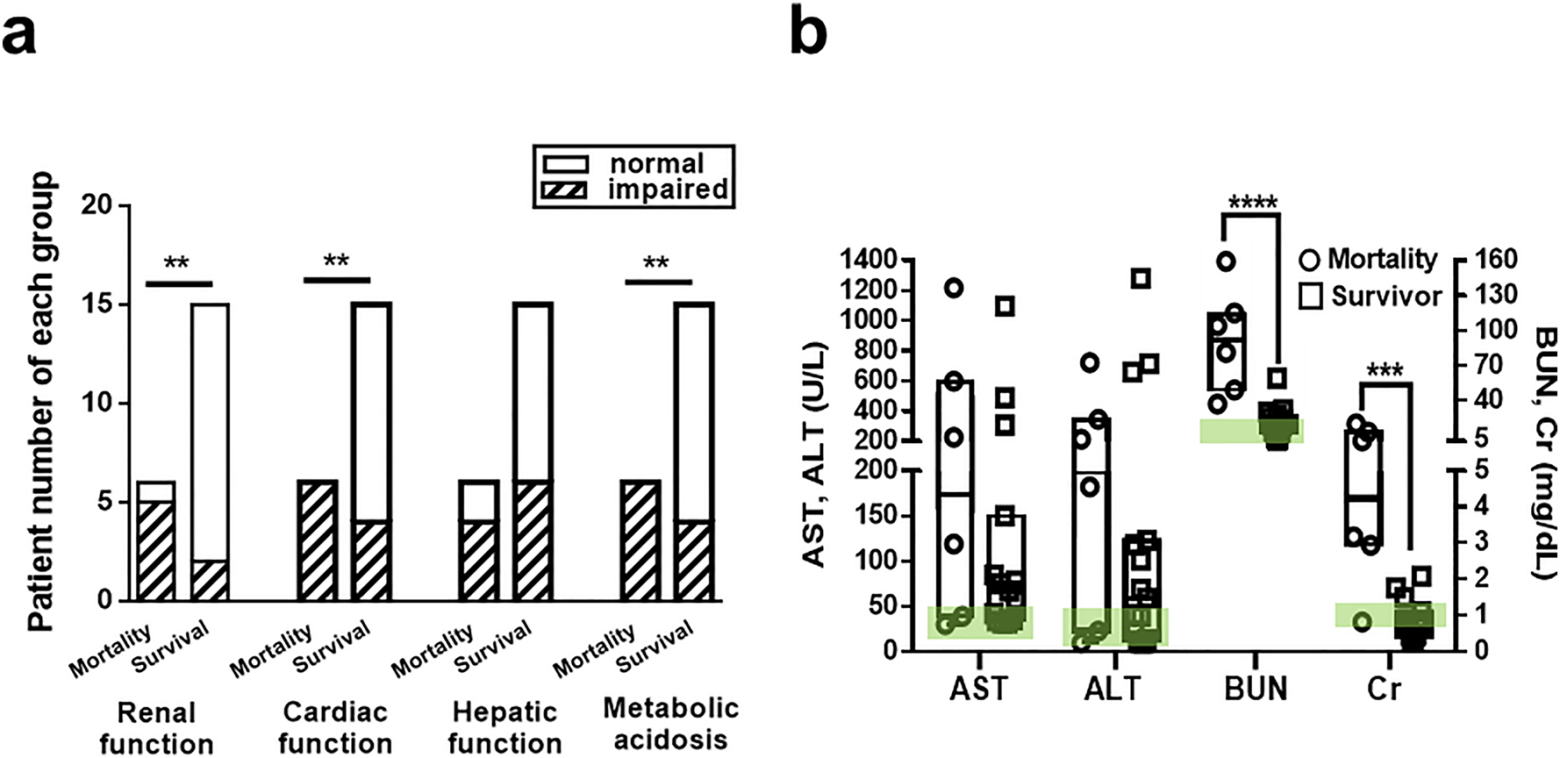
Multisystem complications in patients with SRSE. (**a**) Numbers of patients with renal, cardiac, or hepatic dysfunction or metabolic acidosis in the mortality and survival groups. (**b**) Comparisons of AST, ALT, BUN, and Cr in both groups. **p < 0.01. *SRSE* super-refractory status epilepticus, *BUN* blood urea nitrogen, *Cr* creatinine, *AST* aspartate aminotransferase, *ALT* alanine aminotransferase. Dark lines indicate median values, and boxes indicate interquartile ranges; the green band presenting normal ranges for BUN, Cr, AST, ALT, respectively in (**b**).

The numbers of multi-organ complications were significantly higher in deceased patients than in survivors, such as cardiac dysfunction (*p* = 0.004), renal dysfunction (*p* = 0.006), and metabolic acidosis (*p* = 0.004). Most patients of the M group were affected by metabolic acidosis (100.0%), as well as cardiac (100.0%) and renal dysfunction (83.3%) (Fig. 4a). The renal dysfunction (M vs. S, median, IQR: blood urea nitrogen (BUN, mg/dL) 92, 49–115 vs. 18, 12–27, *p* = 0.0001, creatinine (Cr, mg/dL) 4.2, 2.9–13 vs. 0.68, 0.44–1.1, *p* = 0.0007) and liver dysfunction (M vs. S, aspartate aminotransferase, (AST, U/L) 173, 39–598 vs. 75, 35–150, *p* = 0.415, alanine aminotransferase (ALT, U/L) 198, 23–345 vs. 59, 13–123, *p* = 0.433, Mann–Whitney U test; Fig. 4b) were observed in both groups; renal functions were significantly worse in the M group compared with S group. Cardiac arrhythmia occurred in M group presenting as atrioventricular block, unstable sinus bradycardia, ventricular tachycardia, or cardiac arrest. One patient died of propofol infusion syndrome with metabolic acidosis and sudden cardiac arrest 8 h after IV propofol administration (Fig. 4).

## Discussion

High mortality rates were found in patients with SRSE in this (28.6%) and other (30–42%) cohort studies^7–10,16^. However, factors associated with mortality are not well defined. Clinical features, SE etiology and severity parameters, treatments, and responses between non-survivors and survivors in a homogenous cohort of patients with SRSE from a tertiary teaching hospital were compared to fill this gap. This study showed that deceased patients were relatively younger than survivors and without known systemic diseases or epilepsy. A trend to higher proportions of infection or autoimmune meningoencephalitis with CSF abnormalities in deceased patients was observed compared to surviving patients. The SRSE patients in our cohort with neither identifiable etiology nor pre-existing epilepsy can be considered the new-onset refractory status epilepticus (NORSE)^17^, for which autoantibodies mediated immune etiology might be underestimated across the long recruitment period. Baseline characteristics, initial consciousness levels measured using the GCS, and STESS-based general assessments of SE severity showed no significant differences between the two study groups. However, all deceased patients presented with generalized convulsive SE, which was a significantly higher percentage than that in survivors. Generalized convulsive SE is a more severe SE type than focal convulsive or nonconvulsive SE, which may have caused adverse neurological sequelae, leading to poor outcomes.

There was no significant difference between the two groups in the initial emergency treatment regarding first-line benzodiazepine, IV ASM, and midazolam use after admission. Both mortality and survival groups in our SRSE cohort received adequate treatment at the early, established, and initial refractory stages of SE. The mortality and survival groups differed regarding the numbers and durations of second-line anesthetic use. The use of anesthetics to initiate a therapeutic coma for treating patients with SE has been debated for its benefits and risks regarding treatment outcomes^13,14^. Younger patients with fewer comorbidities presenting with refractory generalized convulsive SE are likely to be treated with more aggressive interventions, including second-line anesthetics, as shown in this study.

Nevertheless, therapeutic coma was found not to change overall mortality but increase the length and costs of hospital stay of patients with SE^18^. Patients with SE treated with anesthetic drugs subsequently have infections, septicemia, and a 2.9-fold relative risk of mortality independent of other possible confounders^19,20^. These studies indicate the adverse effects of anesthetics in treating patients with SE. However, no study has addressed issues specific to SRSE populations. Regarding anesthetics used in patients with SRSE, our data revealed the important finding that an increase in the number and duration of second-line anesthetics is associated with mortality, with a cutoff of 1.5 for mortality discrimination. Multi-organ and systemic complications are adverse effects of prolonged anesthetic use, such as cardiac or respiratory depression, hypotension, metabolic disorders, and paralytic ileus^21^. In line with these findings, our data revealed that renal and cardiac failures and metabolic acidosis were present in the mortality group with increased use of second-line anesthetics. Treating SRSE patients with anesthetics poses a critical dilemma regarding therapeutic needs, benefits, and adverse effects. The poor response to multiple lines of treatment, including benzodiazepines, IV ASMs, and midazolam, usually drives the therapeutic need for more or longer use of second-line anesthetics, which may cause multisystem complications leading to unfavorable outcomes. When adding a second-line anesthetic to midazolam to treat SRSE, the cutoff point of 1.5 suggests that it is favorable to use no more than one add-on anesthetic to reduce mortality. The duration of each anesthetic should be shortened, and prolonged combined use of anesthetics should be avoided to prevent complications. Poor seizure control in patients with SRSE could be attributed to seizure severity, etiologies, underlying physical conditions, ASM and anesthetic treatments susceptibility. Immunotherapy can alleviate the refractory SE for patients with SRSE or NORSE with suspected autoimmune etiologies.

### Limitations

This study had limitations, including the small sample size, making it impossible to perform logistic regression and determine the odds ratios for mortality risk factors. The retrospective cohorts were collected over a long period. The diagnosis and treatment consensus may have varied across time, such as the evolutions of the definitions for convulsive and nonconvulsive SE from ILAE^2^ and Salzburg Consensus Criteria^22^, the autoimmune etiology identification and the related immunotherapy. However, we reviewed the cohort with identical criteria to uncover mortality-associated factors. A retrospective study of a small population of pediatric patients with SRSE collected over a long period (20 patients from 2009 to 2019) showed similar limitations^23^. The varied impacts of SE severity and etiology on mortality could not be fully excluded in this cohort^24^. Based on the study results, the mechanisms underlying unsuccessful tapering of anesthetics that might contribute to SRSE mortality remain not well understood. Anesthetic withdrawal seizure is one of the possibilities considered when breakthrough seizures occur during or following weaning of anesthetics when treating refractory SE^21^, although other etiologies remain to be investigated. Further guidelines on the therapeutic approaches for patients with SRSE are warranted^23^, particularly regarding anesthetic use^14,25^ and the strategies if anesthetic tapering fails^26^. Due to limitations in pharmacological treatment, alternative therapeutic options such as neuromodulation^27–31^, immunotherapy^17,32^, ketogenic diet^33,34^, and hypothermia are potential fields for the unmet needs to treat patients with SRSE^35,36^.

## Conclusions

This 13-year tertiary teaching hospital-based cohort study identified several factors associated with mortality in patients with SRSE, including generalized convulsive SE, failure of anesthetic tapering-off, increased number and duration of second-line anesthetic use, and multiple systemic complications. Increased numbers and treatment durations of second-line anesthetics were significantly associated with mortality in patients with SRSE. It is favorable to avoid exceeding one anesthetic in addition to midazolam to prevent systemic complications and mortality. The patient’s general condition and the therapeutic goal should be frequently re-evaluated to adjust the management. Once adverse effects emerge, the second-line anesthetic should be tapered to avoid complications.

## Supplementary Information

Supplementary Information.

## Data Availability

The datasets generated and analyzed during the current study are not publicly available because the use of medical data is restricted by the IRB. But data are available from the corresponding author on reasonable request.
