# Supplementary figures and images for: Factors associated with mortality in patients with super-refractory status epilepticus
Source: Sci Rep. 2022 Jun 11;12:9670. doi: 10.1038/s41598-022-13726-9 (PMC9188563; doi:10.1038/s41598-022-13726-9)

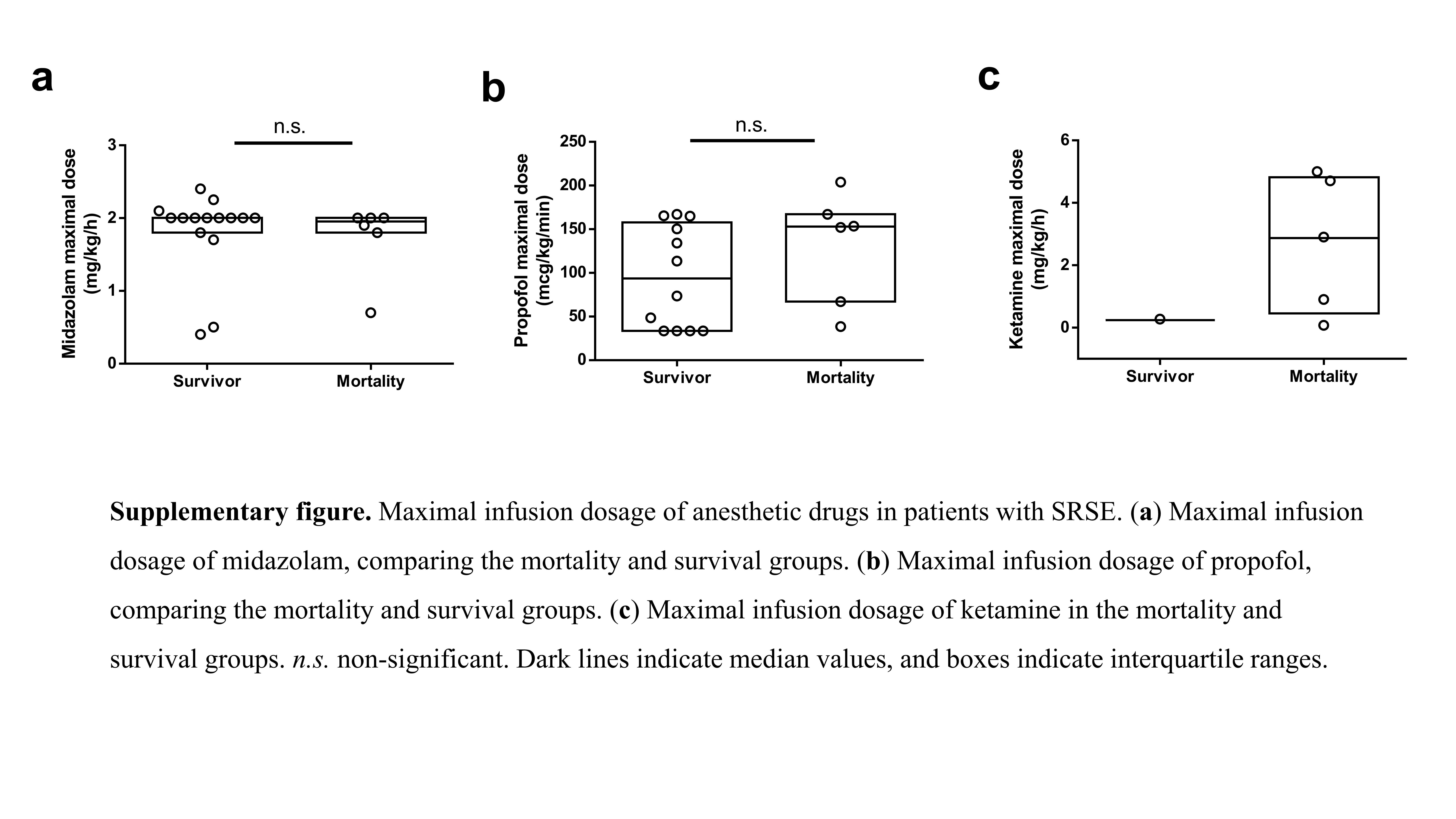

Supplement: Supplementary file 1 — Supplementary Information. [file 41598_2022_13726_MOESM1_ESM.tif]
